# RNA-directed DNA Methylation

**DOI:** 10.1371/journal.pgen.1009034

**Published:** 2020-10-08

**Authors:** Robert M. Erdmann, Colette L. Picard

**Affiliations:** 1 Center for Learning Innovation, University of Minnesota Rochester, Rochester, Minnesota, United States of America; 2 Department of Molecular, Cell, and Developmental Biology, University of California, Los Angeles, California, United States of America

## Abstract

RNA-directed DNA methylation (RdDM) is a biological process in which non-coding RNA molecules direct the addition of DNA methylation to specific DNA sequences. The RdDM pathway is unique to plants, although other mechanisms of RNA-directed chromatin modification have also been described in fungi and animals. To date, the RdDM pathway is best characterized within angiosperms (flowering plants), and particularly within the model plant *Arabidopsis thaliana*. However, conserved RdDM pathway components and associated small RNAs (sRNAs) have also been found in other groups of plants, such as gymnosperms and ferns. The RdDM pathway closely resembles other sRNA pathways, particularly the highly conserved RNAi pathway found in fungi, plants, and animals. Both the RdDM and RNAi pathways produce sRNAs and involve conserved Argonaute, Dicer and RNA-dependent RNA polymerase proteins.

RdDM has been implicated in a number of regulatory processes in plants. The DNA methylation added by RdDM is generally associated with transcriptional repression of the genetic sequences targeted by the pathway. Since DNA methylation patterns in plants are heritable, these changes can often be stably transmitted to progeny. As a result, one prominent role of RdDM is the stable, transgenerational suppression of transposable element (TE) activity. RdDM has also been linked to pathogen defense, abiotic stress responses, and the regulation of several key developmental transitions. Although the RdDM pathway has a number of important functions, RdDM-defective mutants in *Arabidopsis thaliana* are viable and can reproduce, which has enabled detailed genetic studies of the pathway. However, RdDM mutants can have a range of defects in different plant species, including lethality, altered reproductive phenotypes, TE upregulation and genome instability, and increased pathogen sensitivity. Overall, RdDM is an important pathway in plants that regulates a number of processes by establishing and reinforcing specific DNA methylation patterns, which can lead to transgenerational epigenetic effects on gene expression and phenotype.

## Biological functions of RdDM

**Fig 1:**
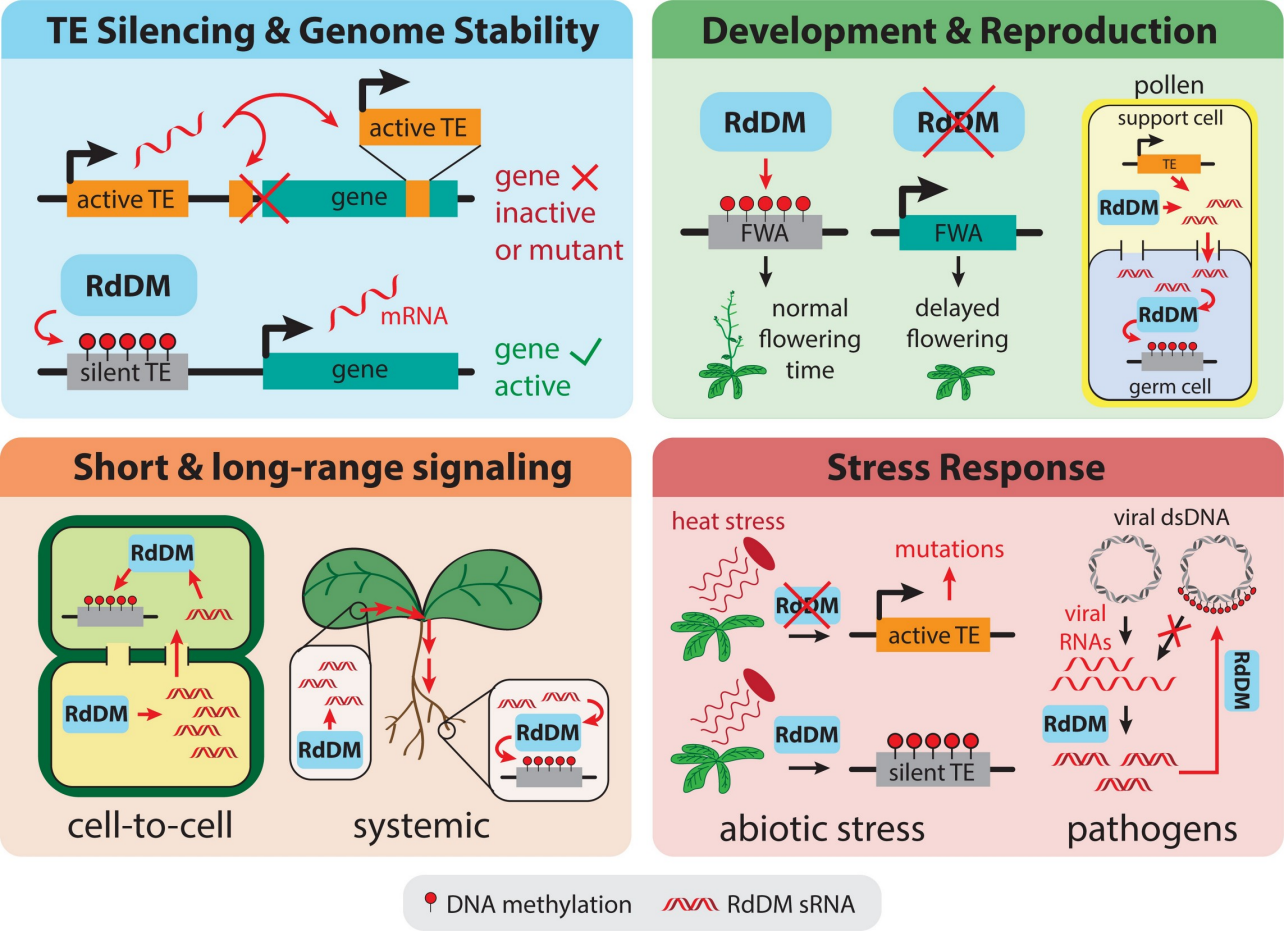
High level overview of several of the biological functions of RdDM. Top left: TE silencing by RdDM prevents TE activation and transposition. Without RdDM, active TEs are free to transpose into genes or promoters, which can disrupt gene expression or result in a mutant protein. Top right: RdDM is involved in several aspects of development; for example, RdDM affects flowering time by repressing FWA. In pollen, TEs become activated in a support cell, leading to the production of sRNAs for RdDM that move to the germ cell in order to reinforce TE silencing. Bottom left: sRNAs involved in RdDM are mobile, and can move between cells through plasmodesmata or systemically via the vasculature, so RdDM-mediated silencing can spread from its point of origin to distal tissues. Bottom right: RdDM is involved in several abiotic stress responses including the heat shock response, and can silence TEs that would otherwise become active and transpose under heat stress. RdDM is also involved in pathogen defense, and can silence viral DNA (either as a viral minichromosome, shown, or as an integrated provirus) using sRNAs derived from viral mRNAs.

RdDM is involved in a number of biological processes in the plant, including stress responses, cell-to-cell communication, and the maintenance of genome stability through TE silencing. An overview of some of the biological functions performed by RdDM is shown in Fig 1.

### Transposable element silencing and genome stability

TEs are pieces of DNA that, when expressed, can move around the genome through a copy-and-paste or cut-and-paste mechanism. New TE insertions can disrupt protein coding or gene regulatory sequences, which can harm or kill the host cell or organism [1]. As a result, most organisms have mechanisms for preventing TE expression. This is particularly key in plant genomes, which are often TE-rich. Some plant species, including important crops like maize and wheat, have genomes consisting of upwards of 80% TEs [1,2]. RdDM plays a key role in silencing these mobile DNA elements in plants by adding DNA methylation over new TE insertions and constantly reinforcing DNA methylation over existing TEs, inhibiting transposition and maintaining long-term genome stability [3]. Although the RdDM mechanism itself is unique to plants, using DNA methylation to silence TEs is a common strategy among eukaryotes [4].

RdDM primarily targets small TEs and TE fragments near genes, which are usually in open, accessible euchromatic regions of the genome that are permissive for gene expression [3,5]. In these regions, the ‘active’ chromatin state has a tendency to spread from expressed genes to nearby repressed regions, like TEs, which can cause these TEs to become activated and transpose [3]. Continuous activity by RdDM opposes the spread of active chromatin, maintaining a silent, repressive heterochromatic state over TEs in these otherwise euchromatic regions. In turn, RdDM activity recruits other pathways that help establish and propagate the silent, heterochromatic state (see 'Interactions between RdDM and other chromatin modifying pathways'). Because of the self-reinforcing nature of these silencing pathways, excessive RdDM activity can also cause the silent, heterochromatic chromatin state over TEs to spread to nearby genes and repress them, with potentially harmful consequences for the organism [3,5]. Therefore, RdDM activity must be finely tuned to maintain a balance between repressing TEs and allowing expression of nearby genes [3].

In addition to maintaining stable silencing of TEs, RdDM can also initiate transcriptional silencing of foreign DNA, including novel TE insertions, virus-derived sequences, and transgenes (also see 'Biotic stresses' and 'Transgene silencing' below) [6–10]. When TEs integrate near genes, RdDM-mediated silencing of the TEs often affects gene expression [1,3]. However, this is not always deleterious, and can sometimes be overcome by other processes [11], or alter gene expression in ways beneficial to the plant. Over evolutionary time, beneficial TEs can become an important part of the mechanism by which a gene is regulated [1,3]. In one example, the gene *ROS1* lies adjacent to a small helitron TE that is normally methylated by RdDM [12,13]. While DNA methylation is normally associated with transcriptional repression, this is not the case at the *ROS1* locus. Instead, methylation of the helitron TE promotes *ROS1* expression, so *ROS1* expression is lost in mutants of the RdDM pathway that cannot methylate the TE [12,13]. Interestingly, *ROS1* encodes a DNA glycosylase that functions to remove DNA methylation from the genome [14]. The link between *ROS1* expression and RdDM activity at this TE ensures that DNA methylation and demethylation activities remain in balance, helping to maintain DNA methylation homeostasis genome-wide [12,13]. Thus, RdDM-mediated regulation of TEs can lead to beneficial regulatory outcomes.

Some TEs have evolved mechanisms to suppress or escape RdDM-based silencing in order to facilitate their own proliferation, leading to an evolutionary arms race between TEs and their host genomes. In one example, a TE-derived sequence was found to produce sRNAs that trigger post-transcriptional repression of a component of the RdDM pathway, inhibiting RdDM [15]. This sequence may have helped the original TE escape RdDM-based silencing and insert itself into the host genome.

Studying how RdDM targets and represses different types of TEs has led to many major insights into how the RdDM mechanism works. The retrotransposon *EVADÉ* (*EVD*) was one of the first TEs specifically shown to be repressed by RdDM-derived sRNAs [16]. Later work used *EVD* to trace the mechanism by which a novel TE insertion became silenced, revealing an important mechanistic link between post-transcriptional gene silencing and RdDM [9]. Studies of other retrotransposons, including *ONSEN*, which is regulated by both RdDM and heat stress [17,18], and Athila family TEs [10], among many others, have also provided valuable insights into RdDM-mediated TE silencing.

### Development and reproduction

A number of epigenetic changes required for normal development and reproduction in flowering plants involve RdDM. In a well-studied example, RdDM is required for repression of the *FLOWERING WAGENINGEN (FWA)* gene, which allows for proper timing of flowering in Arabidopsis [19]. The *FWA* promoter contains tandem repeats that are usually methylated by RdDM, leading to transcriptional repression [20]. Loss of this methylation re-activates *FWA* expression, causing a late-flowering phenotype [19,20]. The loss of DNA methylation and associated late-flowering phenotype can be stably transmitted to progeny. Since the demethylated *fwa* allele leads to a stable, heritable change in the expression of *FWA* without any change to the DNA sequence, it is a classic example of an epiallele.

Mutations in the RdDM pathway can strongly affect gamete formation and seed viability, particularly in plant species with high TE content like maize and *Brassica rapa*, highlighting the importance of this pathway in plant reproduction [21–23]. During gamete formation, it has been hypothesized, and in some cases shown, that RdDM helps reinforce TE silencing in the germ cells [24,25]. In both pollen and ovules, a support cell undergoes epigenetic reprogramming, losing DNA methylation and other epigenetic marks at a number of loci, including TEs [24,26]. This causes TE re-activation and encourages the production of RdDM-derived sRNAs against these TEs in the support cells. The sRNAs are then thought to move from the support cell to the germ cell in order to reinforce TE silencing in the next generation. This phenomenon has been observed in pollen, but has yet to be shown definitively in the ovule [27,28]. This role for sRNAs in plants resembles the role of piRNAs in germline development in Drosophila and some other animals [29,30]. A similar phenomenon may also occur in roots to preserve TE silencing in important stem cell populations [31].

The RdDM pathway is also involved in regulating imprinted expression at some genes [32]. This unusual parent-of-origin-specific expression pattern occurs at several loci in the endosperm during seed development in flowering plants. A few factors involved in the RdDM pathway are themselves imprinted (favoring expression from the paternal allele) in diverse species, including *A*. *thaliana*, *A*. *lyrata*, *C*. *rubella*, and maize [33–36]. RdDM also plays a role in mediating the gene dosage effects seen in seeds derived from interploid crosses [37,38], though the mechanism for this remains largely unknown.

There is also evidence that RdDM plays a role in several other aspects of plant development, including seed dormancy [39], fruit ripening [40], and other pathways involved in flowering [41]. However, most of these data are correlative, and further study is necessary to understand the role of RdDM in these processes.

### Stress response

#### Abiotic stresses

RdDM helps plants respond to a number of abiotic stresses, such as heat stress, drought, phosphate starvation, salt stress, and others [42]. Many TEs become upregulated under abiotic stress conditions [43,44], and thus one function of RdDM in stress response is to help counter this activation. In one example, the retrotransposon ONSEN is upregulated by heat stress, but normally remains suppressed by RdDM-associated sRNAs and can only transpose efficiently in heat-stressed plants that are also deficient in RdDM [17,18]. More generally, in plants exposed to heat stress, several components of the RdDM pathway become upregulated, and mutations in some components of the RdDM machinery reduce heat tolerance, suggesting RdDM plays an important role during heat stress [45,46]. In addition to regulating TEs under stress conditions, RdDM can also regulate genes in order to trigger appropriate stress responses. Under low humidity, leaves produce fewer stomata due to RdDM-mediated downregulation of two genes involved in stomatal development [47]. Similarly, RdDM becomes downregulated in response to salt stress, and this has been shown to trigger the expression of a transcription factor important in salt stress resistance [48].

#### Biotic stresses

RdDM was initially discovered as a response to infection by viroids [49], and along with RNAi plays an important role in defending the plant against viroids and viruses. The RdDM and RNAi machinery recognize viral RNAs and process them into sRNAs, which can then be used by both pathways to degrade viral RNA (RNAi) and silence viral DNA (RdDM) [50–52]. However, little is known about how the RdDM and RNAi machinery distinguish between viral RNAs and RNAs produced by the host plant. Mutants defective in RdDM and other methylation-deficient mutants are often hypersensitive to viral infection [53,54]. Virus-host interactions are another example of an evolutionary arms race, and many plant viruses encode suppressors of both RdDM and RNAi in an attempt to evade the host plant’s defenses [53,55–57].

RdDM is also involved in protecting the plant from other biotic stresses [50], including bacterial infections [58], fungal infections [59], and predation [60]. Loss of RdDM can have opposing effects on resistance for different pathogens. For example, some RdDM mutants have increased susceptibility to the bacterium *Agrobacterium tumefaciens* [61], but those same mutants have decreased susceptibility to the bacterium *Pseudomonas syringae* [58], highlighting the complexity of the different pathogen defense pathways and their interactions with RdDM [62].

#### Transgene silencing

In addition to naturally-occurring foreign nucleic acid stressors like TEs and viruses, artificially introduced DNA sequences, like transgenes, are also targeted for repression by RdDM [6,63]. Transgenes are widely used in genetics research to study gene function and regulation, and in plant breeding to introduce novel and desirable properties into a plant. Transgene silencing by RdDM and other mechanisms has therefore proved problematic for plant researchers. Efforts to understand how transgenes become silenced have ultimately helped reveal much of what we now know about the RdDM pathway (see 'History and discovery of RdDM'). In one early example, researchers sequentially transformed plants with two different transgenes that shared some of their DNA sequence [64]. They found that transforming the second transgene into the plants led to the first transgene gaining DNA methylation and becoming inactivated [64]. This provided an early clue that there existed a trans-acting, sequence-based mechanism for transcriptional silencing of foreign DNA, later shown to be RdDM.

#### Stress and RdDM-mediated epigenetic ‘memory’

Due to the heritability of DNA methylation patterns in plants, and the self-reinforcing nature of RdDM and other DNA methylation pathways, any DNA methylation changes caused by environmental stressors have the potential to be maintained and transmitted to future generations. This can allow stress-induced DNA methylation changes to act as a ‘memory’ of the stressor and help prime the plant or its progeny to respond more efficiently to the stress if re-exposed [50,65]. For example, RdDM-derived sRNAs against TEs or viruses that have already integrated into the genome and been silenced serve as a 'memory' of those prior infections, protecting against future invasions by similar sequences. There is also evidence that DNA methylation changes due to other stressors, such as salt or heat stress, can persist in the progeny of stressed plants even in the absence of the original stressor [66]. In this study, the persistence of the stress-induced DNA methylation changes required several RdDM-related proteins, suggesting that RdDM was involved in maintaining the stress-altered DNA methylation patterns. In another example, resistance to insect attack was transmitted to progeny via DNA methylation changes, and this inheritance was also dependent on functional sRNA biogenesis pathways [50,60]. Thus, RdDM can potentially alter the plant epigenome in response to stress, and helps maintain these changes to modulate future stress responses in the affected plant and its descendants.

### Short and long-range signaling

The sRNA molecules produced by RdDM and other pathways are able to move between cells via plasmodesmata, and can also move systemically through the plant via the vasculature [67–69]. They therefore have the potential to act as signaling molecules. This has been demonstrated in plants engineered to express green fluorescent protein (GFP) [70]. The GFP protein produced by these plants caused them to glow green under certain light conditions. When tissue from a second plant expressing a sRNA construct complementary to GFP was grafted onto the GFP-expressing plant, the GFP fluorescence was lost: after grafting, the sRNAs being produced in the second plant’s tissues were moving into the tissues of the first, GFP-expressing plant, and triggering silencing of GFP [70]. The same study showed that a subset of these mobile sRNAs were triggering the addition of DNA methylation to the GFP locus via RdDM. Therefore, sRNAs involved in RdDM can act as signaling molecules and trigger the addition of DNA methylation at complementary loci in cells far away from where the sRNAs were originally generated. Since then, studies have shown that sRNAs can move and direct RdDM both from shoot to root and root to shoot, though the silencing effect is more robust when sRNAs move from shoot to root [69–72].

Movement of sRNAs that drive RdDM activity plays an important role in plant development, including during reproduction [23,24,27] and root development [31]. In both cases, sRNA movement seems to function primarily as a way to reinforce DNA methylation and silencing of TEs in developmentally important cell types, like germ cells and stem cells. Silencing TEs and maintaining genome integrity in these cells is particularly important because they give rise to many other cells, all of which will inherit any defects or mutations in the original stem cell or germ cell. sRNA movement is also involved in plant-pathogen interactions: sRNAs can move from infected cells to distal uninfected tissues in order to prime a defense response, though to date this has only been shown for RNAi, not RdDM [73].

## Pathways and mechanisms

This section focuses on the pathways and mechanisms by which RdDM leads to sequence-specific DNA methylation. The pathways presented here were characterized primarily in the model plant *Arabidopsis thaliana*, but are likely similar in other angiosperms. Conservation of RdDM in other plant species is discussed in more detail in 'Evolutionary conservation' below.

### RdDM and DNA methylation context

**Fig 2:**
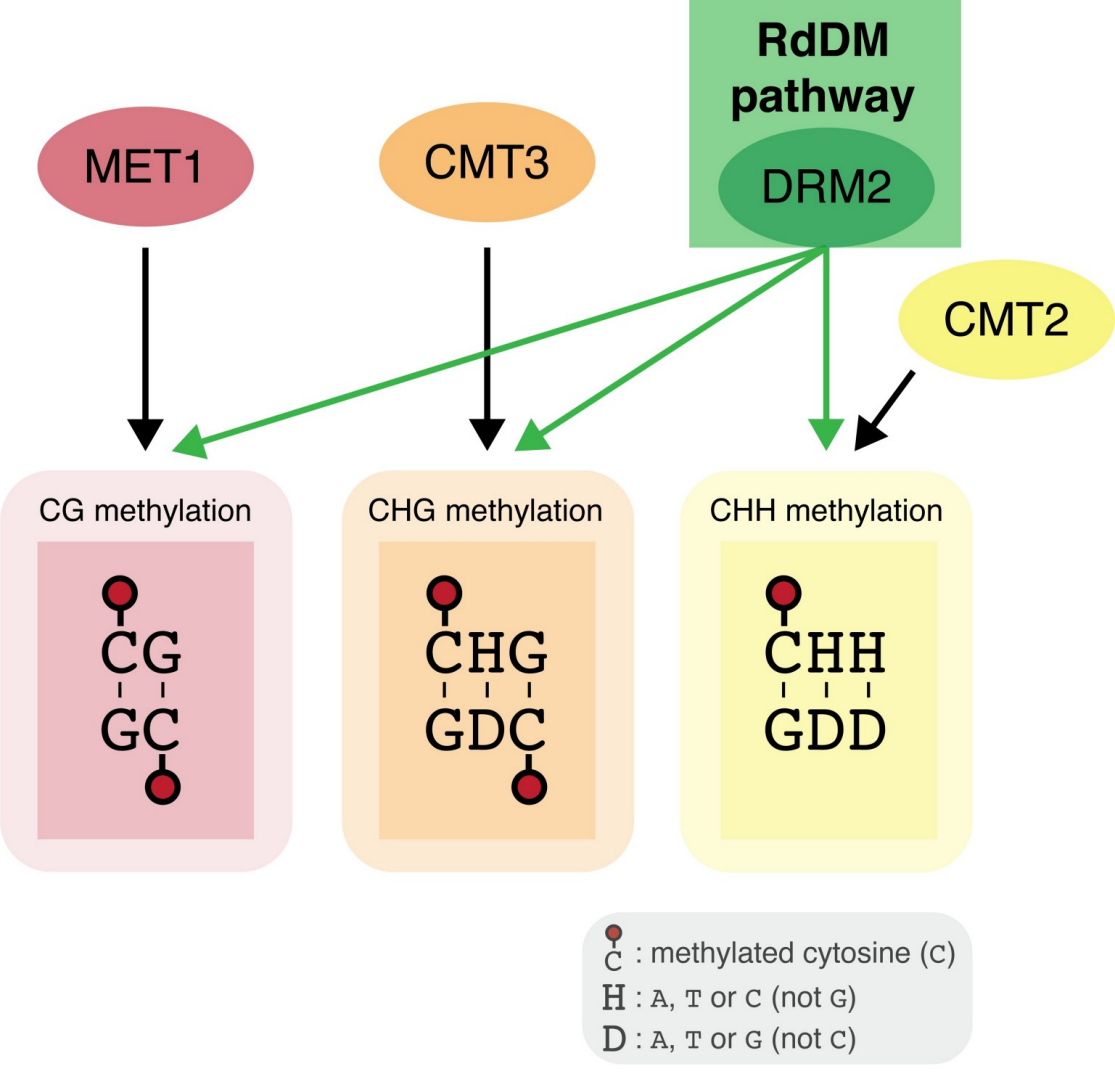
DNA methylation sequence contexts and related DNA methyltransferases. DNA methylation at cytosines followed by guanines (CG methylation) is maintained by MET1, while CHG and CHH methylation are maintained by CMT3 and CMT2, respectively. The methyltransferase involved in RdDM, DRM2, can add DNA methylation regardless of sequence context.

RdDM is the only mechanism in plants that can add DNA methylation to cytosines regardless of sequence context [55]. DNA methylation in plants is typically divided into three categories based on the sequence context of the methylated cytosine: CG, CHG, and CHH, where H is any nucleotide except G (Fig 2). These reflect the different sequence contexts targeted by several DNA methylation pathways in plants. These context-specific pathways are primarily involved in maintaining existing DNA methylation patterns. The highly conserved methyltransferase MET1 (homolog of mammalian DNMT1) maintains DNA methylation in the CG context, while two conserved plant-specific methyltransferases, Chromomethylase 3 (CMT3) and CMT2, help maintain CHG and CHH methylation, respectively [74–77]. Unlike these pathways, RdDM leads to the addition of DNA methylation at all cytosines regardless of their sequence context. Like MET1, CMT2 and CMT3, RdDM is primarily involved in maintaining existing DNA methylation patterns [55]. However, RdDM is also the only pathway capable of adding DNA methylation *de novo* to previously unmethylated regions in plants.

### Overview of the RdDM mechanism

**Fig 3:**
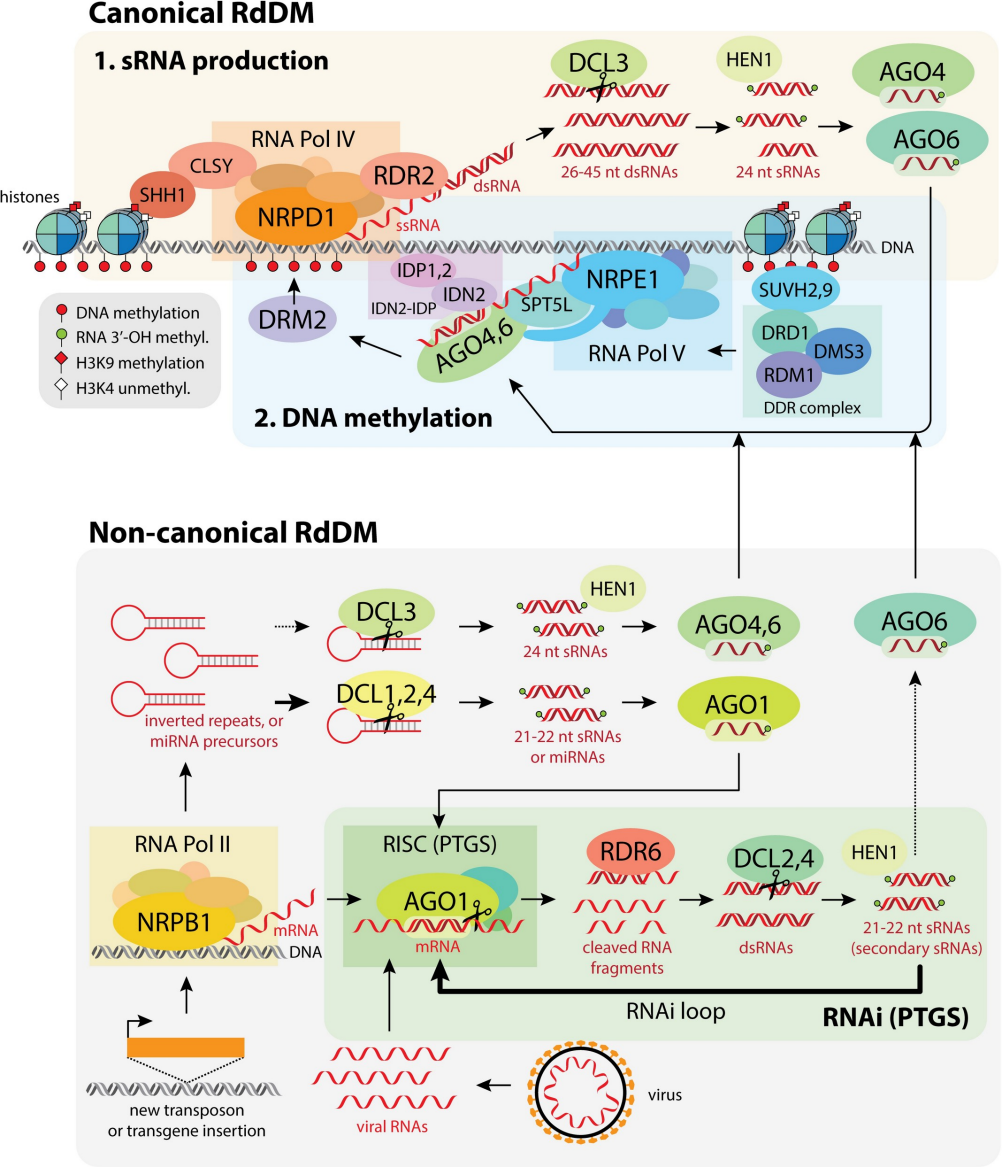
**Schematic of the canonical RdDM pathway (top), and non-canonical RdDM and RNAi/PTGS (bottom).** The canonical RdDM pathway can be broken into (1) sRNA production and (2) targeting DNA methylation to sites of sRNA production. The non-canonical RdDM pathway is closely related to RNAi and other PTGS pathways, and differs from canonical RdDM primarily in the source of sRNAs and sRNA processing. H3K9 = lysine 9 on histone H3; H3K4 = lysine 4 on histone H3; ssRNA = single-stranded RNA; dsRNA = double-stranded RNA, miRNA = microRNA.

The RdDM pathway can be split up into two main processes: the production of sRNAs, and the recruitment of DNA methylation machinery by those sRNAs to specific target loci in the DNA (Fig 3, top) [55,78,79]. These two activities together comprise RdDM, and ultimately lead to DNA methylation being added to cytosines at specific target loci.

### Canonical RdDM

The canonical RdDM pathway is, as its name suggests, the most well-characterized RdDM pathway to date. Canonical RdDM is preferentially recruited to regions that are already DNA-methylated and heterochromatic, and acts to reinforce existing DNA methylation patterns at these loci, forming a positive feedback loop [55,79]. Canonical RdDM makes up the majority of RdDM activity in a cell [79]. The major steps of the canonical pathway are outlined below and in Fig 3 (top), while the various factors involved in the RdDM pathway are described in more detail in Table 1.

**Table 1:** Factors involved in RdDM.

Factor(s)	Factor type	Pathway	Role in RdDM	Known direct interactors	Description	References
**NRPD1 and the Pol IV complex**	RNA polymerase	Canonical RdDM	sRNA production	CLSY proteins, RDR2	Pol IV is a plant-specific RNA polymerase complex and NRPD1, its largest subunit, is specific to the complex. Through its interaction with the CLSY proteins and SHH1, Pol IV is recruited to heterochromatic regions (specifically to H3K9me2- and H3K4me0-containing chromatin), and transcribes single-stranded RNAs precursors of the sRNAs used in the canonical RdDM pathway.	[80,81,93,94]
**NRPE1 and the Pol V complex**	RNA polymerase	All RdDM	DNA methylation of target loci		Pol V is a plant-specific RNA polymerase complex and NRPE1, its largest subunit, is specific to the complex. Pol V transcribes non-coding RNAs that serve as scaffolds for several other RdDM components, most importantly the AGO-sRNA duplex, but also SPT5L, and the IDN2-IDP complex. Both NRPE1 and SPT5L contain an AGO hook motif that helps recruit AGO4 to Pol V transcripts. Mutating the AGO hook motifs on both proteins results in reduced DNA methylation at RdDM target loci, resembling nrpe1 null mutant phenotypes. Binding of the AGO-sRNA duplex to complementary sites along the Pol V transcript leads to recruitment of DRM2 and addition of DNA methylation to target loci.	[80,81,93–95]
**RDR2**	RNA-dependent RNA polymerase	Canonical RdDM	sRNA production	Pol IV	Exists in a complex with Pol IV and converts the nascent Pol IV transcript to double-stranded RNA, which can then be processed by DCL3 to generate sRNAs for canonical RdDM.	[80,83]
**RDR6**	RNA-dependent RNA polymerase	PTGS, non-canonical RdDM	sRNA production		Converts single-stranded RNAs to double-stranded RNAs for processing into 21–22 nt sRNAs by DCL2 and DCL4. Most of these sRNAs lead to PTGS, but some are loaded into AGO6 and participate in non-canonical RdDM.	[9,80]
**DCL1**	Endoribonuclease	PTGS, non-canonical RdDM	miRNA production, sRNA production		An endoribonuclease that cleaves double-stranded RNA, primarily involved in the production of microRNAs that lead to PTGS via AGO1. Can also catalyze the production of 21 nt sRNAs from mRNAs containing inverted repeats, which can be used in either PTGS or non-canonical RdDM depending on the AGO protein they associate with. The four DCL proteins in *A*. *thaliana* (DCL1,2,3,4) compete for access to dsRNA substrates.	[81,96–98]
**DCL2**	Endoribonuclease	PTGS, Non-canonical RdDM	sRNA production		An endoribonuclease that cleaves double-stranded RNA, resulting in 22 nt sRNAs that can be used in both PTGS and non-canonical RdDM. The four DCL proteins in *A*. *thaliana* (DCL1,2,3,4) compete for access to dsRNA substrates, and DCL2,4 can substitute for loss of DCL3 for most RdDM targets.	[80,96,97,99]
**DCL3**	Endoribonuclease	Canonical RdDM	sRNA production		An endoribonuclease that cleaves double-stranded RNA, resulting in 24 nt sRNAs used in canonical RdDM. Preferentially targets the short dsRNAs produced by Pol IV-RDR2, but can also slice other dsRNA substrates, including mRNAs containing inverted repeats or miRNA precursors. The four DCL proteins in *A*. *thaliana* (DCL1,2,3,4) compete for access to dsRNA substrates, and DCL2,4 can substitute for loss of DCL3 for most RdDM targets. When PTGS pathways via DCL2,4 become saturated, DCL3 can step in and process the DCL2,4 dsRNA substrates, triggering a switch from PTGS to RdDM-mediated TGS.	[9,80,96,97,99]
**DCL4**	Endoribonuclease	PTGS, Non-canonical RdDM	sRNA production		An endoribonuclease that cleaves double-stranded RNA, resulting in 21 nt sRNAs that can be used for both PTGS and non-canonical RdDM. The four DCL proteins in *A*. *thaliana* (DCL1,2,3,4) compete for access to dsRNA substrates, and DCL2,4 can substitute for loss of DCL3 for most RdDM targets.	[96,97,99]
**AGO4**	Argonaute protein	Canonical RdDM	DNA methylation of target loci	NRPE1, SPT5L	The main Argonaute protein involved in canonical RdDM. AGO4 is partially redundant with AGO6, which can also function in this pathway, as well as with AGO9 in reproductive tissues. It binds the 24 nt sRNAs produced by the pathway to form an AGO4-sRNA duplex, which can recognize sequences complementary to the sRNA. Assisted by interactions with SPT5L, NRPE1, and the IDN2-IDP complex, the AGO4-sRNA duplex binds a single-stranded, noncoding RNA produced by Pol V, and helps recruit DRM2 to the DNA.	[80,93,100]
**AGO6**	Argonaute protein	All RdDM	DNA methylation of target loci		An argonaute protein that can function in either canonical or non-canonical RdDM pathways. Partially redundant with AGO4 (the main canonical RdDM AGO). Can associate with either 24 nt or 21–22 nt sRNAs to trigger RdDM at complementary loci. By interacting with both 21–22 nt and 24 nt sRNAs, AGO6 helps in the transition from PTGS (normally mediated by 21–22 nt sRNAs) to stable silencing by RdDM (normally mediated by 24 nt sRNAs). Expressed particularly in the root and shoot meristems, which are the two main stem cell populations in plants. This may indicate that plants increase surveillance for novel TEs in order to ensure genome integrity in the key cells that will give rise to most of the other cells in the plant.	[10,80,93,100,101]
AGO9	Argonaute protein	Canonical RdDM	DNA methylation of target loci		A highly specialized AGO expressed primarily in the germline, where it is required for proper female gamete formation. Interacts with 24 nt sRNAs to silence TEs in the germline, similar to the role of PIWI Argonaute proteins in animals.	[25,100,102]
**AGO1**	Argonaute protein	PTGS, non-canonical RdDM	sRNA production		Binds microRNAs or 21–22 nt sRNAs, which it uses to recognize complementary sequences on other RNAs. When an AGO1-sRNA duplex (often called the RISC) finds a complementary single-stranded mRNA, the RNA is cleaved by AGO1, destroying the mRNA and causing PTGS. The resulting RNA fragments can then be converted to dsRNAs by RDR6 and processed by DCL2,4 to form secondary 21–22 nt sRNAs. These are predominantly loaded back into AGO1, forming a self-reinforcing ‘RNAi loop’ (Fig 3). However, some of the 21–22 nt sRNAs are loaded into AGO6 instead, leading to RdDM.	[80,91,97,100]
**DRM2**	DNA methyltransferase	All RdDM	DNA methylation of target loci		The main DNA methyltransferase involved in RdDM. Catalyzes the addition of a methyl group to cytosines in DNA. Recruited by the AGO4-sRNA duplex after it binds to a complementary sequence in a Pol V transcript, but the mechanism by which this happens is not well understood.	[80,103]
**SHH1/DTF1**	DNA and chromatin binding protein	Canonical	sRNA production	CLSY1	Required for Pol IV-derived sRNA production at a subset of RdDM loci. Via its SAWADEE domain, SHH1 binds histone H3 with specific modifications associated with heterochromatin and DNA methylation: methylation of the 9th lysine (H3K9me2) and unmethylated K4 (H3K4me0). By interacting with SHH1 via the CLSY proteins, Pol IV is recruited to heterochromatic/silent chromatin. To date, SHH1 has only been shown to directly interact with CLSY1. The ability of SHH1 to associate with Pol IV/NRPD1 is mostly abolished in clsy1,2 double mutants, so recruitment of Pol IV by SHH1 likely requires CLSY proteins.	[104,105,106,107]
**CLSY1, CLSY2**	putative chromatin remodelers	Canonical	sRNA production	Pol IV, SHH1	Required for SHH1 interaction with and recruitment of Pol IV to a subset of target loci. Mutually exclusive with loci regulated by CLSY3 and CLSY4. Together, the four CLSY proteins regulate nearly all Pol IV-derived sRNAs, and loss of all four results in a near total loss of 24-nucleotide sRNA production. Requires H3K9me2, likely through interaction with SHH1. sRNAs regulated by CLSY1,2 are enriched in the chromosome arms, while those regulated by CLSY3,4 are enriched in the pericentromere.	[107,108]
**CLSY3, CLSY4**	putative chromatin remodelers	Canonical	sRNA production, Pol IV targeting	Pol IV	Involved in recruitment of Pol IV to a subset of target loci. Mutually exclusive with loci regulated by CLSY1 and CLSY2. Together, the four CLSY proteins regulate nearly all Pol IV-sRNAs, and loss of all four results in a near total loss of 24-nucleotide sRNA production. sRNAs regulated by CLSY3,4 are enriched in the pericentromere, while sRNAs regulated by CLSY1,2 are enriched in the chromosome arms.	[107,108]
**HEN1**	RNA methylase	Both	sRNA production	none	Stabilizes sRNAs by adding methylation to the 3'-OH groups.	[109]
**SUVH2, SUVH9**	methyl-DNA binding proteins	Both	DNA methylation of target loci	DDR complex, MORC1, MORC6	A pair of closely related methyl-DNA binding proteins that interact with the DDR complex and are required for proper localization of the DDR complex and Pol V. By recruiting Pol V to regions with DNA methylation, which tend to be silent, heterochromatic regions, SU(VAR)3-9 homolog (SUVH) 2 and 9 help form a positive feedback loop that reinforces RdDM-mediated silencing. May also associate with MORCs.	[110]
**DDR complex (RDM1, DMS3, DRD1)**	putative chromatin remodeling complex	Both	DNA methylation of target loci	SUVH2, SUVH9	The DDR complex, composed of DRD1, DMS3, and RDM1, is thought to facilitate access of Pol V to its target sites, possibly by unwinding DNA downstream of Pol V. Interacts with SUVH2,9, which bind methylated DNA, and this interaction may help recruit Pol V to regions of existing heterochromatin. RDM1 also binds single-stranded DNA, which may help unwind the DNA to facilitate recruitment of DRM2.	[88,110–113]
**SPT5L/RDM3/KTF1**	transcription factor	Both	DNA methylation of target loci	AGO4, Pol V transcripts	Interacts with AGO4 and helps recruit it to the RNA scaffold produced by Pol V. Like the Pol V subunit NRPE1, SPT5L contains an AGO hook motif in its C-terminal domain. The motifs on both NRPE1 and SPT5L redundantly help recruit AGO4 to loci being transcribed by Pol V. Mutating the AGO hook motifs on both proteins results in reduced DNA methylation at RdDM target loci, resembling nrpe1 null mutant phenotypes. Also required for co-transcriptional slicing of Pol V transcripts.	[95,114,115]
SWI/SNF complex	chromatin remodeling complex	Both	DNA methylation of target loci	IDN2	The Switch/Sucrose non-fermentable (SWI/SNF) complex is a chromatin remodeling complex that is recruited to Pol V scaffolds by the IDN2-IDP complex, where it affects nucleosome positioning. SWI/SNF may promote RdDM by making the chromatin more accessible, which may facilitate access of DRM2 to DNA.	[116]
**IDN2-IDP complex**	dsRNA-binding protein	Both	DNA methylation of target loci	SWI/SNF complex	A complex composed of IDN2 and IDP1 (also called IDNL1) or IDP2 (IDNL2). IDN2, and possibly IDP1, can bind the dsRNA duplex formed when AGO-associated sRNAs hybridize with the Pol V scaffold. This complex is thought to help stabilize base pairing between the AGO-sRNA and Pol V scaffold RNA. IDN2-IDP may also facilitate recruitment of the SWI/SNF complex to Pol V scaffolds. Additionally, IDP1 can bind unmethylated DNA, which may help recruit DRM2 to regions lacking DNA methylation.	[116–118]
NERD	GW repeat- and PHD finger-containing protein	Non-canonical RdDM	sRNA production, DNA methylation of target loci	AGO2	Forms a non-canonical RdDM pathway that includes a number of genes involved in PTGS, including AGO2. Binds histone H3 and AGO2. Required for 21 nt sRNA accumulation at some non-canonical RdDM targets, including novel TE insertions. Leads to histone tail modifications associated with transcriptional repression; because these modifications can recruit other DNA methylation machinery, including canonical RdDM, it is unclear if the effect of NERD on DNA methylation is direct or indirect.	[79,92]
MORC1, MORC6	GHKL ATPases	Both	DNA methylation of target loci (?)	SUVH2, SUVH9, IDN2, DMS3	Microrchidia 1 (MORC1) and MORC6 form a heterodimer and may interact with the DDR complex to recruit Pol V. However, they are thought to mainly act downstream of DNA methylation to promote silencing. Their precise role in RdDM is still unclear.	[80,90,110]
DRM1	DNA methyltransferase	All RdDM	DNA methylation of target loci		A homolog of DRM2 that is only expressed during sexual reproduction, specifically in the egg cell and potentially the early embryo. DRM2 is likely the main RdDM methyltransferase in all other tissues.	[119]
HDA6	Histone deacetylase	Canonical RdDM	sRNA production		May facilitate Pol IV recruitment by creating a permissive chromatin state for SHH1 binding by removing histone acetylation, promoting H3K9 methylation. In *histone deacetylase 6* (*hda6*) mutant plants, HDA6 target loci lose Pol IV targeting and sRNA biogenesis, suggesting HDA6 is involved in Pol IV recruitment at a subset of RdDM target loci. Further, normal Pol IV targeting cannot be restored after re-introduction of functional HDA6, suggesting that HDA6 is also required to propagate the trans-generational 'memory' of where Pol IV should be targeted. HDA6 physically associates with MET1 and facilitates CG methylation maintenance by MET1, which may also be important for sRNA production at HDA6-dependent loci.	[80,120]

#### sRNA production

The first part of the RdDM pathway revolves around the biogenesis of sRNAs. A plant-specific RNA polymerase complex, RNA Polymerase IV (Pol IV), is first recruited to silent heterochromatin via its interaction with CLASSY (CLSY) proteins and SAWADEE homeodomain homolog 1 (SHH1) (also see 'Interactions between RdDM and other chromatin modifying pathways' below) [79–81]. Pol IV transcribes these regions to produce short single-stranded RNAs (ssRNAs) roughly 30 to 45 nucleotides in length, each of which is the precursor for a single sRNA [82–84]. These ssRNAs are converted into double-stranded RNAs (dsRNAs) co-transcriptionally by RNA-directed RNA polymerase 2 (RDR2), which physically associates with Pol IV [83]. The dsRNAs are then cleaved by the endoribonuclease Dicer-like 3 (DCL3) into 24 nucleotide (nt) sRNAs. Pol IV, RDR2, and DCL3 alone are sufficient for the production of 24 nt sRNAs *in vitro* [84], suggesting that while other factors involved in this part of the pathway may help increase efficiency or specificity, they are not required for Pol IV-mediated sRNA production.

While nearly all 24 nt sRNAs involved in RdDM are produced through the Pol IV-RDR2-DCL3 pathway, a small proportion are produced through other pathways. For example, some RNA Polymerase II (Pol II) transcripts that contain an inverted repeat sequence form double-stranded hairpin structures that can be directly cleaved by DCL3 to form 24 nt sRNAs (Fig 3, bottom) [79,85].

#### DNA methylation of target loci

In the second part of the pathway, the RdDM DNA methylation machinery is guided to DNA sequences complementary to the sRNAs generated in the first part of the pathway. One strand from each 24 nt double-stranded sRNA is loaded into Argonaute (AGO) proteins AGO4, AGO6, or AGO9 [55]. AGO3 may also be able to function in this pathway [86]. Argonautes are a large, highly conserved family of proteins that can bind sRNAs, forming a protein-sRNA duplex that enables them to recognize and bind other RNA sequences complementary to their sRNA partner [87]. Once formed, the AGO-sRNA duplex finds and binds complementary sequences along an RNA ‘scaffold’ produced by the plant-specific RNA Polymerase V (Pol V), with the help of interactions with Suppressor of Ty insertion 5-like (SPT5L), the Involved in de novo 2—IDN2 Paralog (IDN2-IDP) complex, and the Pol V subunit NRPE1 [88]. This leads to the recruitment of the DNA methyltransferase enzyme Domains Rearranged Methyltransferase 2 (DRM2), which methylates nearby DNA [55,79,89]. The mechanism by which the AGO-sRNA duplex recruits DRM2 is not yet well understood [90].

### Non-canonical RdDM

Recent work has revealed a number of variations of the RdDM pathway, collectively referred to as non-canonical RdDM (Fig 3, bottom) [79]. Unlike canonical RdDM, the non-canonical pathways are generally involved in establishing initial DNA methylation at new target loci, like novel TE insertions, rather than maintaining existing heterochromatin. Actively expressing elements like new TE insertions are normally strongly targeted by post-transcriptional gene silencing (PTGS/RNAi) pathways (Fig 3, bottom). Non-canonical RdDM occurs primarily as a byproduct of these PTGS pathways, leading to the initial establishment of a silent, heterochromatic state over the new TE or other target locus. Once that initial silent state is established, Pol IV can be recruited to the locus by CLSY and SHH1, and the canonical RdDM pathway takes over the long-term maintenance of silencing [79]. Therefore, the non-canonical RdDM pathways often act as a temporary bridge between initial post-transcriptional silencing of novel elements by RNAi, and long-term transgenerational transcriptional silencing via canonical RdDM [9,10,79]. Consistent with this role in initiation of novel silencing, non-canonical RdDM targets relatively few loci in comparison to canonical RdDM [79].

The primary difference between the canonical and non-canonical RdDM pathways lies in the origin and biogenesis of the sRNAs involved. The canonical RdDM pathway involves 24 nt sRNAs, which are specific to that pathway and come predominantly from a single source (the Pol IV-RDR2 complex). In contrast, the non-canonical RdDM pathways involve 21–22 nt sRNAs from a variety of sources, allowing *de novo* DNA methylation to be initiated at many different types of loci. These 21–22 nt sRNAs are not specific to non-canonical RdDM, and also function in other PTGS pathways. In fact, only a small fraction of 21-22nt sRNAs are involved in RdDM, with the majority instead driving a positive feedback loop amplifying the PTGS response (Fig 3) [91]. The functional outcome of a specific 21–22 nt sRNA depends on the AGO protein it ultimately associates with: sRNAs that associate with AGO4, AGO6 or AGO9 result in RdDM and DNA methylation, while sRNAs that associate with other AGOs, like AGO1, primarily result in PTGS [55,79].

By using 21–22 nt sRNAs derived from a variety of sources, non-canonical RdDM can flexibly induce *de novo* DNA methylation and silencing at many different types of loci. One of the primary sources of 21–22 nt sRNAs is Pol II transcripts (Fig 3). Some of these transcripts, particularly those produced from TEs, viruses, or certain non-protein-coding transcripts, are targeted by PTGS pathways like miRNAs or RNAi, leading to cleavage of the transcript. The resulting fragments can be converted into dsRNA by RDR6 and then processed into 21–22 nt sRNAs by DCL2 or DCL4 [8]. Most of these 21–22 nt sRNAs are loaded into AGO1 and feed back into PTGS, amplifying PTGS efficiency [79]. However, some will instead associate with AGO6, leading to RdDM [10]. dsRNAs resulting from RDR6 activity can also sometimes processed by DCL3 instead of DCL2/4 and trigger RdDM [9]. Additionally, some Pol II transcripts contain inverted repeat sequences, which can form double-stranded hairpin-like structures (Fig 3). These can be cleaved by DCL proteins independent of RDRs to produce either 21–22 nt or 24 nt sRNAs that can participate in RdDM [79]. Similarly, miRNA precursors, which also form hairpin structures and are normally cleaved by DCL1 to produce miRNAs, can instead be cleaved by other DCLs to form sRNAs for RdDM [79]. While most non-canonical RdDM occurs via AGO6 or AGO4, there is also a version of the pathway where sRNAs instead associate with AGO2, which together with the NERD complex (Needed for RDR2-independent DNA methylation) recruits DRM2 to target loci and triggers DNA methylation [92]. Since the non-canonical pathways are not yet as well characterized as the canonical RdDM pathway [79], there likely remain additional sources of sRNAs used for RdDM that have not yet been uncovered.

### Factors involved in RdDM

A number of factors involved in RdDM are listed in Table 1, along with additional details about their function and corresponding references. Several factors primarily involved in PTGS (Fig 3) that sometimes participate in RdDM are also listed. Factors in **bold** are shown in Fig 3.

### Interactions between RdDM and other chromatin modifying pathways

Different chromatin states, like active euchromatin or silent heterochromatin, are defined by a combination of specific histone modification and DNA methylation patterns. Repressive chromatin modifications, like DNA methylation, help promote DNA compaction and reduce DNA accessibility, while other modifications help open chromatin and increase accessibility. Methylation of the 9th lysine of histone H3 (H3K9), primarily in the form of H3K9 trimethylation (H3K9me3) in animals and H3K9 dimethylation (H3K9me2) in plants, is a highly conserved repressive modification [121,122]. Lack of H3K4 methylation (H3K4me0) is also associated with repression, along with several other histone modifications and variants. The combination of DNA methylation, H3K9me2, and H3K4me0 is strongly associated with heterochromatin in plants.

Since DNA methylation and repressive histone modifications together define heterochromatin, most DNA methylation pathways in plants recognize and interact with repressive histone marks and vice-versa, forming positive feedback loops that help maintain the repressive chromatin state [123]. The RdDM-associated protein SHH1 recognizes H3K4me0 and H3K9me2 at heterochromatic loci and recruits Pol IV to these loci to trigger additional DNA methylation at these regions [106]. Similarly, SUVH2 and SUVH9 help recruit Pol V to loci with DNA methylation [110]. Thus, both major parts of the canonical RdDM pathway are preferentially recruited to regions that are already in the silent, heterochromatic state marked by DNA methylation, H3K9me2, and H3K4me0. DNA methylation at these same heterochromatic loci is also recognized by the histone methyltransferases SUVH4/KYP, SUVH5, and SUVH6, which bind to non-CG methylation and add H3K9me2 to nearby histones [123,124], closing the positive feedback loop. Similarly, CMT3 and CMT2, the two DNA methyltransferases involved in the maintenance of CHG and CHH methylation respectively (Fig 2) [75], both bind and add DNA methylation to H3K9me2-marked heterochromatin, forming their own feedback loop with SUVH4/5/6 [123,125]. These interactions help strongly reinforce silencing at TEs and other heterochromatic regions.

A similar feedback loop occurs in animals. HP1 plays a vital role in maintaining heterochromatin by propagating H3K9 methylation through a positive feedback loop with the H3K9 methyltransferase SUV39H [126]. H3K9 methylation recruits HP1, which recruits SUV39H to deposit more H3K9 methylation [126]. Though HP1 is conserved in plants, its function in this feedback loop is not conserved [127]. Instead, the positive feedback loops between H3K9me2 and the RdDM and CMT2/3 DNA methylation pathways fulfill a similar function in propagating H3K9me2. More recently, a plant-specific protein, Agenet Domain Containing Protein 1 (ADCP1), was also identified that may function analogously to HP1 in maintaining H3K9me2 levels in heterochromatin, facilitating heterochromatin formation [128].

Ultimately, the constant reinforcement of silencing chromatin modifications at heterochromatic loci creates a repressive chromatin state wherein the DNA and histones (nucleosomes) become tightly packed together. This helps silence gene expression by physically inhibiting access to the DNA, preventing RNA Polymerase II, transcription factors and other proteins from initiating transcription [129]. However, this same compaction also prevents factors involved in heterochromatin maintenance from accessing the DNA, which could lead to the silent, compact state being lost. This is particularly true in the dense constitutive heterochromatin surrounding the centromere. In these regions, the chromatin remodeler DDM1 plays a crucial role in DNA methylation maintenance by displacing nucleosomes temporarily to allow methyltransferases and other factors access the DNA [5,130,131]. However, since most RdDM targets are small TEs in open, accessible and gene-rich regions (see ‘TE silencing and genome stability’), few RdDM sites require DDM1 [5,99]. In fact, dense heterochromatin inhibits RdDM [5]. By contrast, CMT2 and CMT3 preferentially function in constitutive heterochromatin and depend strongly on DDM1 to maintain silencing over these regions [3,5,131]. Similarly, MET1, which maintains DNA methylation at CG sites after replication (Fig 2), requires DDM1 to access heterochromatin and maintain CG methylation in those regions [132]. Thus, DDM1 is a key regulator of DNA methylation in dense heterochromatin, but regulates sites mostly independently from RdDM [5,99].

Interactions between RdDM and the other three maintenance DNA methylation pathways (Fig 2) are limited and predominantly indirect. The DNA methyltransferase MET1 robustly maintains CG methylation genome-wide (Fig 2), including at RdDM target sites. In RdDM mutants, non-CG methylation at RdDM target sites is lost, but CG methylation is still maintained, suggesting that MET1 activity is independent of RdDM [99]. However, although *met1* mutants lose CG methylation as expected, they also lose much of their non-CG methylation, including at RdDM target loci [99]. At these sites, silencing can still be initiated by RdDM in *met1* mutants, but it is not maintained or transmitted to progeny, suggesting that MET1 is important for the maintenance, but not initiation, of silencing at a subset of RdDM target loci [120,133]. This effect is likely indirect: loss of MET1 leads to loss of H3K9me2 at some sites, which inhibits the recruitment of Pol IV and therefore prevents maintenance of DNA methylation via canonical RdDM, although the non-canonical pathways (which do not involve Pol IV) are not affected [99,120]. Loss of the histone deacetylase HDA6, which facilitates maintenance methylation by MET1 at some loci, has a similar effect, suggesting that multiple different factors involved in maintaining heterochromatin likely facilitate RdDM-mediated DNA methylation maintenance [120].

Loss of RdDM leads to strong loss of non-CG methylation at TEs in gene-rich regions in the chromosome arms, but has little effect on DNA methylation levels in the constitutive heterochromatin around the centromere [3,5,99]. This suggests that CMT2 and CMT3, which function primarily to maintain CHG and CHH methylation in dense constitutive heterochromatin, do not depend on RdDM activity [3,5,99]. Similarly, in *cmt2*,*cmt3* double mutants, many TEs in the chromosome arms remain methylated, presumably due to the persistent activity of RdDM, indicating that loss of CMT2/3 has little effect on RdDM activity [3,5]. This suggests that RdDM and CMT2/3 function mostly independently and at distinct loci: RdDM is the main pathway responsible for maintaining non-CG DNA methylation in euchromatic, gene rich regions, while CMT2 and CMT3 maintain non-CG DNA methylation in constitutive heterochromatin. In mutants defective in both RdDM and CMT2/CMT3, all non-CG methylation in the genome is eliminated [74], demonstrating that together RdDM and CMT2/CMT3 account for all non-CG methylation in the genome.

### Balance between DNA methylation and demethylation

Most DNA methylation mechanisms in plants are self-reinforcing (see above), including RdDM: Pol IV and Pol V are both recruited to heterochromatic regions that already have DNA methylation, encouraging additional DNA methylation via canonical RdDM [55]. Positive feedback loops like these can cause DNA methylation activity to spread out from the intended methylated target sites into genes or other regulatory elements, which can negatively affect gene expression. To prevent this spreading, DNA methylation pathways are opposed by passive and active DNA demethylation. DNA methylation can be lost passively with each cell division, because newly-synthesized strands of DNA lack DNA methylation until it is re-added by one of the maintenance DNA methylation pathways [134]. DNA methylation can also be actively removed in plants by DNA glycosylases, which remove methylated cytosines via the base excision repair pathway. In Arabidopsis, there are four proteins responsible for removing DNA methylation: Repressor of silencing 1 (ROS1), Demeter (DME), Demeter-like 2 (DML2), and Demeter-like 3 (DML3) [135,136]. These DNA glycosylases help prevent the spread of DNA methylation from RdDM targets to active genes [14,137]. Loss of active DNA demethylation in *ros1;dml2;dml3* triple mutants leads to a widespread increase in DNA methylation levels, whereas ectopic expression of ROS1 leads to progressive loss of DNA methylation at many loci [138], highlighting the importance of balancing DNA methylation and demethylation activity.

Interestingly, expression of the DNA demethylase ROS1 is directly tied to RdDM activity: DNA methylation over a TE targeted by RdDM in the *ROS1* promoter is required for *ROS1* expression [12,13], though other factors are also involved in regulating *ROS1* [139,140]. Since *ROS1* expression is tied to DNA methylation at a specific TE, *ROS1* expression is strongly reduced in plants with defective RdDM that lose the ability to methylate that TE [12]. This general mechanism helps maintain DNA methylation homeostasis by tuning DNA demethylation activity to DNA methylation activity, helping to ensure that DNA methylation patterns can be stably maintained over time.

## Evolutionary conservation

### Origins of RdDM pathway members

While all eukaryotes share three RNA polymerases (RNA Pol I, II and III), plants have two additional polymerases, Pol IV and Pol V. Both Pol IV and V share an evolutionary origin, deriving from Pol II [94,141]. In other eukaryotic kingdoms that lack these two specialized RNA polymerases, Pol II transcribes the precursors of small RNAs used in silencing pathways–in fact, Pol II transcripts are also sometimes processed into sRNAs in plants (Fig 3). It has been hypothesized that the origin of both Pol IV and Pol V is rooted in “escape from adaptive conflict” [142]. The idea is that potential tensions between the “traditional” function of Pol II and the small RNA biogenesis function could be relieved by duplication of Pol II and subfunctionalization of the resulting multiple RNA polymerases.

**Fig 4:**
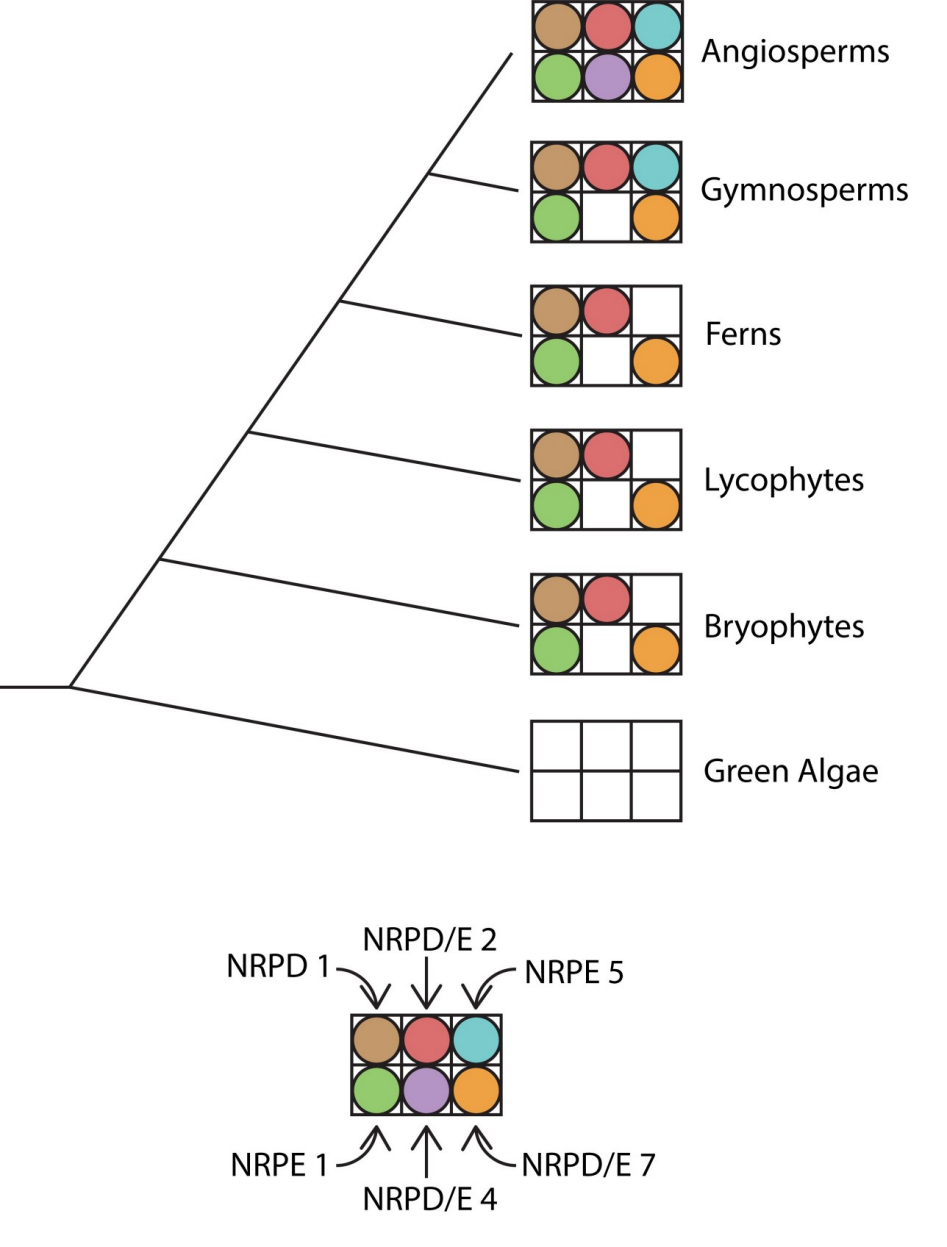
A schematic depicting the evolutionary conservation of selected Pol IV and V subunit orthologs within the plant kingdom. Subunits beginning with NRPD are Pol IV subunits, subunits beginning with NRPE are Pol V subunits, and subunits labeled as NRPD/E are found in both Pol IV and V. [141] A filled circle for a subunit indicates that an ortholog for that subunit has been identified within the associated lineage.

Analyses of evolutionary lineage for Pol IV and Pol V are complicated to some extent by the fact that each enzyme is actually comprised of at least 12 subunits [141]. In *Arabidopsis thaliana*, some subunits are shared between Pol IV and Pol V, some are unique to each polymerase, and some are shared between Pol II, IV, and V [143]. Orthologs of certain Pol IV and V subunits have been found in all lineages of land plants, including ferns, liverworts, and mosses (Fig 4) [142,144]. These findings argue for a shared origin of Pol IV and V dating back to early land / vascular plants.

Much of the work done to elucidate the genes and proteins involved in the RdDM pathway has been performed in *Arabidopsis thaliana*, a model angiosperm. However, studies of Pol IV and V conducted in maize show some key differences with Arabidopsis. Maize Pol IV and V differ from each other in terms of only one subunit (the largest one). In Arabidopsis, Pol IV and V differ from each other in terms of three subunits [145]. However, maize utilizes a set of interchangeable catalytic subunits–two in the case of Pol IV and three in the case of Pol V–that provide additional specialization of polymerase functionality [145]. While differences exist, overall there is a broad overlap in RdDM functions and components between the different angiosperm species studied to date.

**Fig 5:**
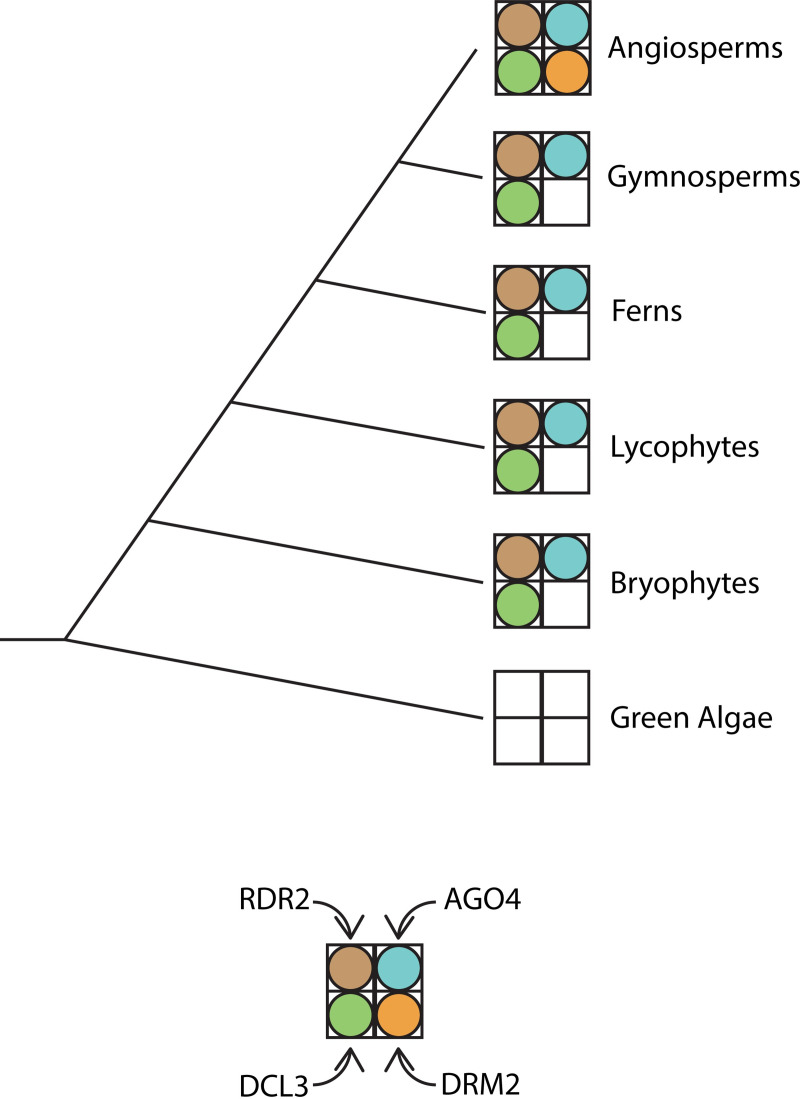
A schematic depicting the evolutionary conservation of selected RdDM pathway component orthologs within the plant kingdom. A filled circle for a subunit indicates that an ortholog for that subunit has been identified within the associated lineage.

Outside of Pol IV and Pol V, a large proportion of key RdDM component proteins (for example, DCL3 and AGO4) have orthologs found within each class of land plants, which provides support for the hypothesis that some form of the RdDM pathway evolved early within the plant lineage (Fig 5) [142]. However, RdDM pathway functionality does appear to change to an appreciable extent between different plant species and lineages. For example, while gymnosperms have functional Pol IV and produce 24 nt small RNAs, the biogenesis of sRNAs within gymnosperms is much more heavily skewed towards 21 nt than 24 nt sRNAs [146]. This suggests that canonical RdDM may be rarer or less pronounced in gymnosperms than in angiosperms. Similarly, while orthologs of DRM2 are found in various angiosperms, there are no known DRM2 orthologs in other plant lineages [147]. One possibility is that angiosperms have the “most complete” version of the RdDM pathway, with all other plant lineages possessing robust and functional subsets of the pathway. However, since nearly all of the work on RdDM has been done in angiosperms, it is also possible that alternative versions of RdDM in other lineages have simply not yet been uncovered, particularly if these alternative versions include different proteins or proteins without clear homologs in angiosperms.

### Relationships with sRNA silencing pathways in other kingdoms

All eukaryotic kingdoms host some form of small RNAs. One such class of sRNAs is the Piwi-interacting RNAs (piRNAs). Much like in RdDM, piRNAs primarily function to target and silence transposons, particularly in the germline [29,30]. However, piRNAs are only found in animals, are longer than the small RNAs functioning in RdDM (24–32 nucleotides), and mediate their functions through interactions with a different subclass of AGO proteins, the PIWI subfamily, which are absent from plants [29,30]. MicroRNAs (miRNAs) are another class of small RNA with silencing properties [148]. While miRNAs are in a similar size range as RdDM sRNAs (~21 nt), miRNAs associate with a distinct set of Argonaute proteins that silence target RNAs by initiating their degradation or blocking their downstream translation into proteins, rather than recruiting DRM2 to add DNA methylation to nearby DNA. Both RdDM and the miRNA pathways involve related proteins from the Argonaute and Dicer families [148].

Perhaps the most analogous pathways to RdDM in another eukaryotic kingdom are the sRNA directed transcriptional gene silencing (TGS) and co-transcriptional gene silencing (CTGS) pathways in *Schizosaccharomyces pombe* [149]. In *S*. *pombe*, TGS directs methylation of H3K9, leading to heterochromatin formation, and is directed by sRNAs produced from the targeted regions [150]. Similar to canonical RdDM, this pathway is a positive feedback loop: sRNAs are generated preferentially from heterochromatin-rich areas of the genome, and these sRNAs direct the addition of K3K9 methylation to maintain/spread heterochromatin. Meanwhile, CTGS is directed by AGO1-bound sRNAs, similar to PTGS within plants, and results in the inhibition of transcription by Pol II, as well as to Pol II release [151,152]. Unlike RdDM, TGS and CTGS in *S*. *pombe* do not rely on transcription from non-Pol II sources or lead to the addition of DNA methylation. However, the *S*. *pombe* pathways and RdDM share many of the same components, like RNA-directed RNA polymerases and sRNAs, and have similar functions in maintaining heterochromatin.

## History and discovery of RdDM

Introducing transgenes into organisms has been a widely used tool in plant genetics research for decades. However, researchers often find that their introduced transgenes are not expressed as strongly as expected, or sometimes even at all, a phenomenon called transgene silencing [153]. The discovery of transgene silencing in the 1990s spurred a great deal of interest in understanding the mechanisms behind this silencing [154–156]. Researchers found that transgene silencing was ubiquitous, occurring in multiple species (including Arabidopsis, Tobacco, and Petunia), and was associated with increased DNA methylation over and around the silenced transgene [157–159].

Around the same time in 1994, work in tobacco plants had revealed a new pathway involving RNAs that resulted in DNA methylation. Researchers found that when viroids were introduced into the plant and integrated into the plant genome, the viroid sequences, but not the host genome, gained DNA methylation [49]. The deposition of methylation over these foreign viroid sequences helped inhibit viroid replication, and was therefore thought to represent a plant pathogen defense mechanism. The evidence suggested that the viroid RNAs produced during viroid replication were being used by the plant as a template to help target DNA methylation to the viroid sequences. This mechanism was therefore named RNA-directed DNA methylation, or RdDM [49].

RdDM turned out to be the solution to the transgene mystery: like viroids and viruses, transgenes are foreign sequences, and as a result they are often recognized as foreign invaders and targeted for silencing by RdDM and PTGS. Since transgene silencing was a reliable marker of RdDM activity, researchers were able to design genetic screens to identify mutants that failed to trigger silencing at transgenes, reasoning that these genes were likely to be involved in the RdDM pathway. These experiments revealed many parts of the pathway, including RNA Pol IV and V, Dicer-like proteins, Argonautes, and others [6,160,161].

The involvement of sRNAs in RdDM was initially suspected due to the similarity between RdDM and RNAi, the latter of which had recently been shown to involve small RNAs [49,162]. To test whether sRNAs were involved in RdDM, RNA hairpin structures complementary to a specific gene promoter were introduced into Arabidopsis and Tobacco [163]. The hairpin RNAs were processed into sRNAs, which were able to trigger the addition of DNA methylation to the targeted promoter and silence the gene [163]. This demonstrated that sRNAs could direct DNA methylation to specific loci. Later efforts showed that the sRNAs involved in RdDM were approximately 24–26 nt long, while the sRNAs associated with RNAi were only about 21–22 nt in length [164,165]. Soon after, the identification of AGO4 and characterization of its role in RdDM led to predictions, later confirmed, that 24 nt sRNAs were associating with AGO4 and directing DNA methylation to complementary loci [165,166].

Early work on transgene silencing and RdDM also identified SDE4 as required for the production of most sRNAs involved in RdDM [167]. SDE4 would later be identified as the largest subunit of Pol IV, and renamed NRPD1. A number of studies published in quick succession from multiple research groups, utilizing both forward and reverse genetic approaches, went on to identify and characterize Pol IV and Pol V as highly specialized plant RNA polymerases involved in RdDM [168–171]. The Pol IV / Pol V naming convention was adopted shortly thereafter [88,141].

## Potential biotechnology applications of RdDM

Since the mechanism underlying the sequence-specificity of RdDM is well known (Fig 3), RdDM can be ‘tricked’ into targeting and silencing endogenous genes in a highly specific manner, which has a number of potential biotechnological and bioengineering applications. Several different methods can be used to trigger RdDM-based DNA methylation and silencing of specific genes. One method, called virus-induced gene silencing (VIGS), involves inserting part of the promoter sequence of the desired target gene into a virus [172]. The virus will reproduce the chunk of promoter sequence as part of its own RNA, which is otherwise foreign to the plant. Because the viral RNA is foreign, it will be targeted for PTGS and processed into sRNAs, some of which will be complementary to the original target gene’s promoter (Fig 3). A subset of these sRNAs will recruit the RdDM machinery to the target gene to add DNA methylation. In one study, researchers used this method with an engineered Cucumber Mosaic Virus to recruit RdDM to silence a gene that affected flower pigmentation in petunia, and another that affected fruit ripening in tomato [173]. In both cases, they showed that DNA methylation was added to the locus as expected. In petunia, both the gain of DNA methylation and changes in flower coloration were heritable, while only partial silencing and heritability were observed in tomato. VIGS has also been used to silence the *FWA* locus in Arabidopsis, which resulted in plants that flowered later than normal [172]. The same study also showed that the inhibitory effect of VIGS on *FWA* and flowering can become stronger over the course of successful generations [172].

Another method to target RdDM to a desired target gene involves introducing a hairpin RNA construct that is complementary to the target locus. Hairpin RNAs contain an inverted repeat, which causes the RNA molecule to form a double-stranded RNA (dsRNA) structure called an RNA hairpin. The dsRNA hairpin can be processed by DCL proteins into sRNAs which are complementary to the target locus, triggering RdDM at that locus (Fig 3). This method has been used in several studies [12,174,175].

Changes induced by RdDM can sometimes be maintained and inherited over multiple generations without outside intervention or manipulation, suggesting that RdDM can be a valuable tool for targeted epigenome editing. Recent work has even bypassed RdDM altogether by artificially tethering DRM2 (or other components of the RdDM pathway) directly to specific target loci, using either zinc finger nucleases or CRISPR [90,176]. In these experiments, tethering the RdDM machinery to a specific locus led to gain of DNA methylation at the target site that was often heritable for multiple generations, even once the artificial construct was removed through crossing. For all of these methods, however, more work on minimizing off-target effects and increasing DNA methylation efficiency is needed.

Genetically Modified Organisms (GMOs) have played a large role in recent agricultural research and practice, but have proven controversial, and face regulatory barriers to implementation in some jurisdictions. GMOs are defined by the inclusion of “foreign” genetic material into the genome. The treatment of plants with engineered RNAs or viruses intended to trigger RdDM does not change the underlying DNA sequence of the treated plant’s genome; only the epigenetic state of portions of the DNA sequence already present are altered. As a result, these plants are not considered GMOs. This has led to efforts to utilize RdDM and other RNA-mediated effects to induce agriculturally-beneficial traits, like altering pathogen or herbicide susceptibility, or speeding up plant breeding by quickly inducing favorable traits [177–179]. However, while this is an area of active interest, there are few broadly implemented applications as of now.

## Supporting information

S1 Text Version history of the text file. (XML)

S2 Text Peer reviews and response to reviews. (XML)
